# Two Functional Fatty Acyl Coenzyme A Ligases Affect Free Fatty Acid Metabolism To Block Biosynthesis of an Antifungal Antibiotic in Lysobacter enzymogenes

**DOI:** 10.1128/AEM.00309-20

**Published:** 2020-05-05

**Authors:** Kaihuai Li, Rongxian Hou, Huiyong Xu, Guichun Wu, Guoliang Qian, Haihong Wang, Fengquan Liu

**Affiliations:** a College of Plant Protection, Nanjing Agricultural University, Nanjing, China; b Key Laboratory of Integrated Management of Crop Diseases and Pests (Nanjing Agricultural University), Ministry of Education, Nanjing, China; c Institute of Plant Protection, Jiangsu Academy of Agricultural Sciences, Nanjing, China; d Guangdong Provincial Key Laboratory of Protein Function and Regulation in Agricultural Organisms, College of Life Sciences, South China Agricultural University, Guangzhou, Guangdong, China; Shanghai Jiao Tong University

**Keywords:** heat-stable antifungal factor (HSAF), *Lysobacter enzymogenes* OH11, fatty acyl-CoA ligase (FCL), intracellular free fatty acids

## Abstract

In Lysobacter enzymogenes OH11, RpfB1 and RpfB2 were predicted to encode acyl coenzyme A (CoA) ligases. RpfB1 is located in the Rpf gene cluster. Interestingly, we found an RpfB1 homolog (RpfB2) outside this canonical gene cluster, and nothing is known about its functionality or mechanism. Here, we report that *rpfB1* and *rpfB2* can functionally replace EcFadD in the Escherichia coli *fadD* mutant JW1794. RpfB activates long-chain fatty acids (n-C_16:0_ and n-C_18:0_) for the corresponding fatty acyl-CoA ligase (FCL) activity *in vitro*, and Glu-361 plays critical roles in the catalytic mechanism of RpfB1 and RpfB2. Deletion of *rpfB1* and *rpfB2* resulted in significantly increased heat-stable antifungal factor (HSAF) production, and overexpression of *rpfB1* or *rpfB2* completely suppressed HSAF production. Deletion of *rpfB1* and *rpfB2* resulted in increased *L. enzymogenes* diffusible signaling factor 3 (*Le*DSF3) synthesis in *L. enzymogenes*. Overall, our results showed that changes in intracellular free fatty acid levels significantly altered HSAF production. Our report shows that intracellular free fatty acids are required for HSAF production and that RpfB affects HSAF production via FCL activity. The global transcriptional regulator Clp directly regulated the expression of *rpfB1* and *rpfB2*. In conclusion, these findings reveal new roles of RpfB in antibiotic biosynthesis in *L. enzymogenes*.

**IMPORTANCE** Understanding the biosynthetic and regulatory mechanisms of heat-stable antifungal factor (HSAF) could improve the yield in Lysobacter enzymogenes. Here, we report that RpfB1 and RpfB2 encode acyl coenzyme A (CoA) ligases. Our research shows that RpfB1 and RpfB2 affect free fatty acid metabolism via fatty acyl-CoA ligase (FCL) activity to reduce the substrate for HSAF synthesis and, thereby, block HSAF production in *L. enzymogenes*. Furthermore, these findings reveal new roles for the fatty acyl-CoA ligases RpfB1 and RpfB2 in antibiotic biosynthesis in *L. enzymogenes*. Importantly, the novelty of this work is the finding that RpfB2 lies outside the Rpf gene cluster and plays a key role in HSAF production, which has not been reported in other diffusible signaling factor (DSF)/Rpf-producing bacteria.

## Abstract (precis)

Understanding the biosynthetic and regulatory mechanisms of heat-stable antifungal factor (HSAF) could improve the yield in Lysobacter enzymogenes. Here, we report that RpfB1 and RpfB2 encode acyl coenzyme A (CoA) ligases. Our research shows that RpfB1 and RpfB2 affect free fatty acid metabolism via fatty acyl-CoA ligase (FCL) activity to reduce the substrate for HSAF synthesis and, thereby, block HSAF production in *L. enzymogenes*. Furthermore, these findings reveal new roles for the fatty acyl-CoA ligases RpfB1 and RpfB2 in antibiotic biosynthesis in *L. enzymogenes*. Importantly, the novelty of this work is the finding that RpfB2 lies outside the Rpf gene cluster and plays a key role in HSAF production, which has not been reported in other diffusible signaling factor (DSF)/Rpf-producing bacteria.

## INTRODUCTION

Lysobacter enzymogenes strain OH11, originally isolated from the rhizosphere of green pepper, is a nonpathogenic strain that was used to control crop fungal diseases (1–4). Heat-stable antifungal factor (HSAF), a polycyclic tetramate macrolactam, is a newly identified broad-spectrum antifungal antibiotic isolated from the biocontrol species *L. enzymogenes* and is regarded as an attractive agent for the biological control of fungal diseases of agriculturally important plants (5–8). HSAF exhibits inhibitory activity against a wide range of fungal species, and its chemical structure and mode of action are distinct from those of existing antifungal drugs or fungicides (1, 7). Therefore, the identification of genes and growth conditions that regulate HSAF production is of significant interest.

**FIG 1:**
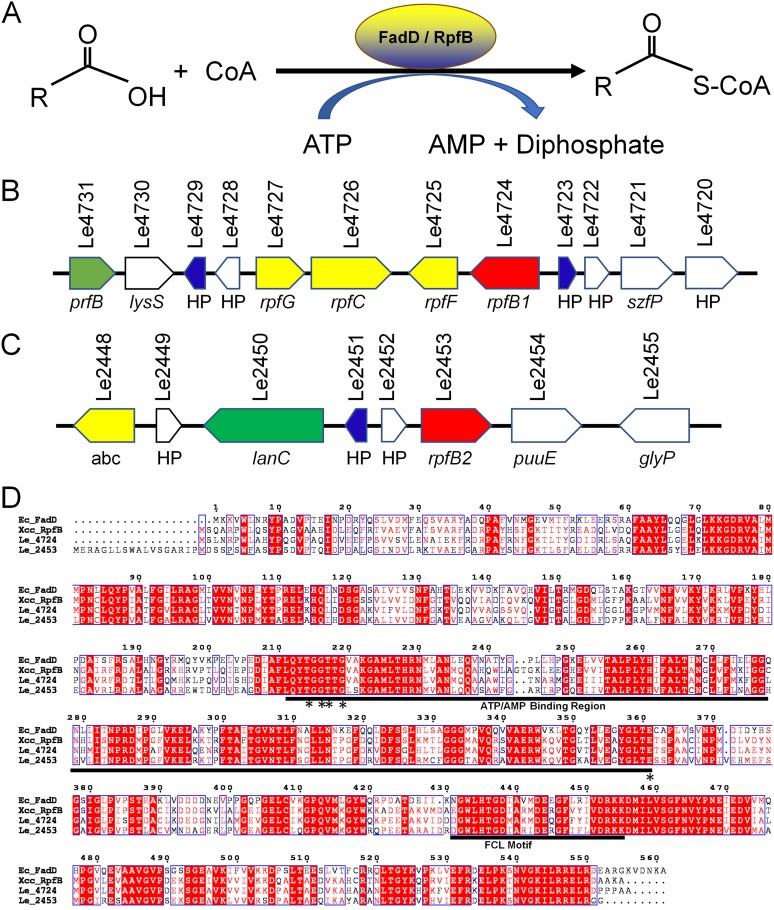
Identification and sequence characterization of RpfB in *L. enzymogenes* OH11. (A) Chemical equation of the acyl-CoA ligase reaction. (B) Chromosomal region of *L. enzymogenes* surrounding the *rpfB1* gene. *prfB*, peptide chain release factor 2; *lysS*, lysyl-tRNA synthetase; HP, hypothetical protein; *rpfG*, two-component system, response regulator; *rpfC*, two-component system, sensor histidine kinase; *rpfF*, enoyl-CoA hydratase/isomerase family; *rpfB1*, long-chain fatty acid-CoA ligase; *szfP*, SWIM zinc finger protein. (C) Chromosomal region of *L. enzymogenes* surrounding the *rpfB2* gene. *abc*, ABC transporter, ATP-binding protein; *lanC*, lanthionine synthetase C-like protein; *rpfB2*, long-chain fatty acid-CoA ligase; *puuE*, 4-aminobutyrate aminotransferase; *glyP*, glyoxalase family protein. (D) Alignment of *L. enzymogenes*, X. campestris, and E. coli RpfBs. The alignment was performed with Clustal W based on identical residues. The ATP/AMP and FCL motifs are underlined. The sites that were experimentally confirmed in E. coli are denoted by asterisks.

The diffusible signaling factor (DSF) represents a new class of widely conserved quorum sensing (QS) signals that regulate various biological functions in pathogenic and beneficial environmental bacteria (9, 10). The Rpf gene cluster is important for the DSF signaling network in bacteria, and the role of RpfF and RpfB in this gene cluster in DSF production and turnover has been well documented (11–13). A previous study revealed that *L. enzymogenes* diffusible signaling factor 3 (*Le*DSF3)-mediated QS positively regulates the biosynthesis of HSAF in *L. enzymogenes* (10, 14). However, whether RpfB can also affect HSAF synthesis in *L. enzymogenes* remains unknown. RpfB is annotated as a gene encoding a fatty acyl coenzyme A (CoA) ligase (FCL), also known as fatty acid CoA synthetase (15), which plays a crucial role in fatty acid β-oxidation to yield acyl-CoAs for membrane lipid synthesis in bacteria (Fig. 1A) (16). RpfB acts strongly on short- (C_8_), medium- (C_10_ to C_14_), and long-chain (C_16_ to C_18_) fatty acids, which indicates that this protein is a broad chain-length FCL. In contrast, RpfB FCL activity was very low with DSF fatty acid substrates (15).

Two RpfB homologs encoding an FCL were identified in the genome of *L. enzymogenes* OH11. Interestingly, there exists an RpfB homolog (Le2453) outside the canonical Rpf gene cluster, and nothing is known about its functionality or mechanistic action. To distinguish these two genes, *rpfB* (Le4724), located immediately upstream of *rpfGCF* within the *rpf* gene cluster, was named *rpfB1*, and the other *rpfB* gene, located upstream of *puuE* (4-aminobutyrate aminotransferase), was named *rpfB2* (Le2453) (Fig. 1B and C). We hypothesized that RpfB can regulate the synthesis of HSAF, but the regulatory mechanism of RpfB remains unknown. Whether the RpfB1 and RpfB2 homologs synergistically or differentially regulate the synthesis of HSAF in *L. enzymogenes* remains unknown.

In the present study, we demonstrated that two long-chain acyl-CoA ligases, namely, RpfB1 and RpfB2, play a role in fatty acid β-oxidation in *L. enzymogenes*. Either RpfB1 or RpfB2 effectively operates in HSAF synthesis by affecting intracellular free fatty acid metabolism in *L. enzymogenes*. The global transcription regulator Clp directly positively regulates RpfB expression.

## RESULTS

### Two conserved *rpfB* genes in the *L. enzymogenes* genome.

To investigate the function of RpfB in *L. enzymogenes* OH11, alignments of RpfB with FadD proteins from Escherichia coli (17) and Xanthomonas campestris (15) were examined (Fig. 1D). The results showed that the RpfB1 protein shares 58% and 75% identity with E. coli FadD and X. campestris RpfB, respectively. We also aligned RpfB2 with E. coli FadD and X. campestris RpfB, and the respective identity values were 58% and 62%, respectively. The RpfB1 protein shares 64% identity with RpfB2 (Fig. 1D). Our sequence alignments (Fig. 1D) showed that the ATP/AMP and FCL signature motifs identified for E. coli FadD and X. campestris RpfB are highly conserved in *L. enzymogenes* RpfB1 and RpfB2 (17, 18). Based on these criteria, it seems reasonable that RpfB1 and RpfB2 could be functional acyl-CoA ligases that play crucial roles in *L. enzymogenes*.

### *L. enzymogenes rpfB1* and *rpfB2* complemented the growth of an E. coli *fadD* mutant strain.

**FIG 2:**
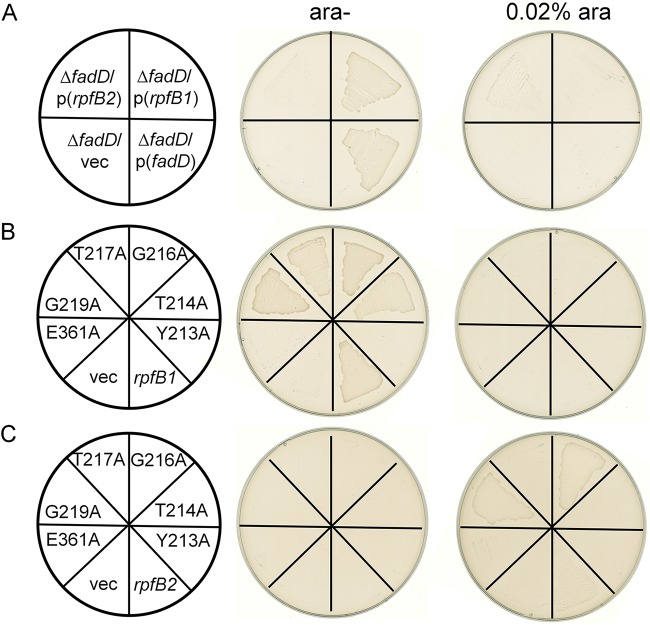
Expression of *L. enzymogenes* RpfBs restored the growth of the E. coli *fadD* mutant JW1794. Transformants of the E. coli *fadD* mutant JW1794 were grown on M9 minimal medium plates with oleic acid as the sole carbon source. Growth was tested in either the presence or absence of arabinose. The strains tested were as follows: (A) JW1794 carrying the plasmid pBAD24M-EcD, pBAD24M-*rpfB1*, or pBAD24M-*rpfB2*, encoding E. coli *fadD* (EcfadD), *L. enzymogenes rpfB1*, or *L. enzymogenes rpfB2*, respectively, or the vector plasmid pBAD24M. (B) Strain JW1794 carrying the plasmid pBAD24M-*rpfB1*, pBAD24M-*rpfB1* Y213A, pBAD24M-*rpfB1* T214A, pBAD24M-*rpfB1* G216A, pBAD24M-*rpfB1* T217A, pBAD24M-*rpfB1* G219A, or pBAD24M-*rpfB1* E361A, encoding *L. enzymogenes rpfB1* or the Y213A, T214A, G216A, T217A, G219A, or E361A *rpfB1* mutant, respectively, or the vector plasmid pBAD24M. (C) Strain JW1794 carrying the plasmid pBAD24M-*rpfB2*, pBAD24M-*rpfB2* Y213A, pBAD24M-*rpfB2* T214A, pBAD24M-*rpfB2* G216A, pBAD24M-*rpfB2* T217A, pBAD24M-*rpfB2* G219A, or pBAD24M-*rpfB2* E361A, encoding *L. enzymogenes rpfB2* or the Y213A, T214A, G216A, T217A, G219A, or E361A *rpfB2* mutant, respectively, or the vector plasmid pBAD24M.

The E. coli JW1794 strain is a *fadD* mutant that cannot survive in minimal medium when exogenous fatty acids are the sole source of carbon (19). This deficiency can be complemented by a plasmid carrying a gene encoding acyl-CoA ligase. To test whether RpfB can function as an acyl-CoA ligase, we expressed *rpfB1* and *rpfB2* in the E. coli Δ*fadD* strain (JW1794). The vector plasmid pBAD24M and an EcFadD complementation vector were used as a negative control and a positive control, respectively. The resulting transformants were tested on M9 medium plates supplemented with oleic acid as the sole carbon source. *rpfB1* functionally complemented the E. coli Δ*fadD* strain and allowed growth on oleic acid as the sole carbon source without arabinose as an inducer, whereas JW1794 carrying *rpfB2* exhibited growth on oleic acid as the sole carbon source but needed arabinose as an inducer (Fig. 2A). The results showed that either *rpfB1* or *rpfB2* functions as an acyl-CoA ligase, which is consistent with the results obtained for X. campestris RpfB (15).

A previous study revealed that E. coli FCL (FadD) contains two sequence elements, namely, an ATP/AMP signature motif and an FCL motif, the residues of which are critical for catalytic activity (17). To test whether this motif was important for catalytic activity in RpfB1 or RpfB2, we substituted the RpfB1 and RpfB2 residues Tyr-213, Thr-214, Gly-216, Thr-217, Gly-219, and Glu-361 of the ATP/AMP signature motif and FCL motif with alanine (Ala) by site-directed mutagenesis. The growth of the E. coli strain JW1794 carrying plasmids encoding these mutant proteins was tested with oleic acid as the sole carbon source on M9 medium plates. Our results showed that point mutations of the Tyr-213 and Glu-361 residues in RpfB1 abolished FCL activity. The strains expressing the RpfB2 Y213A, T214A, T217A, and E361A mutant proteins failed to grow either in the presence or in the absence of arabinose, whereas the strains expressing the RpfB2 G216A and G219A mutant proteins grew better in the presence of arabinose than the strain expressing the RpfB2 wild-type protein (Fig. 2B and C). These results demonstrated that Tyr-213 and Glu-361 are required for RpfB1 FCL activity and that Tyr-213, Thr-214, Thr-217, and Glu-361 are critical for RpfB2 FCL activity. To confirm this phenotype, these strains were grown in M9 broth, and similar results were obtained (see Fig. S1 in the supplemental material). Altogether, these results indicate that RpfB1 and RpfB2 have acyl-CoA ligase activity *in vivo*.

### Biochemical properties of the two *L. enzymogenes* RpfBs.

**FIG 3:**
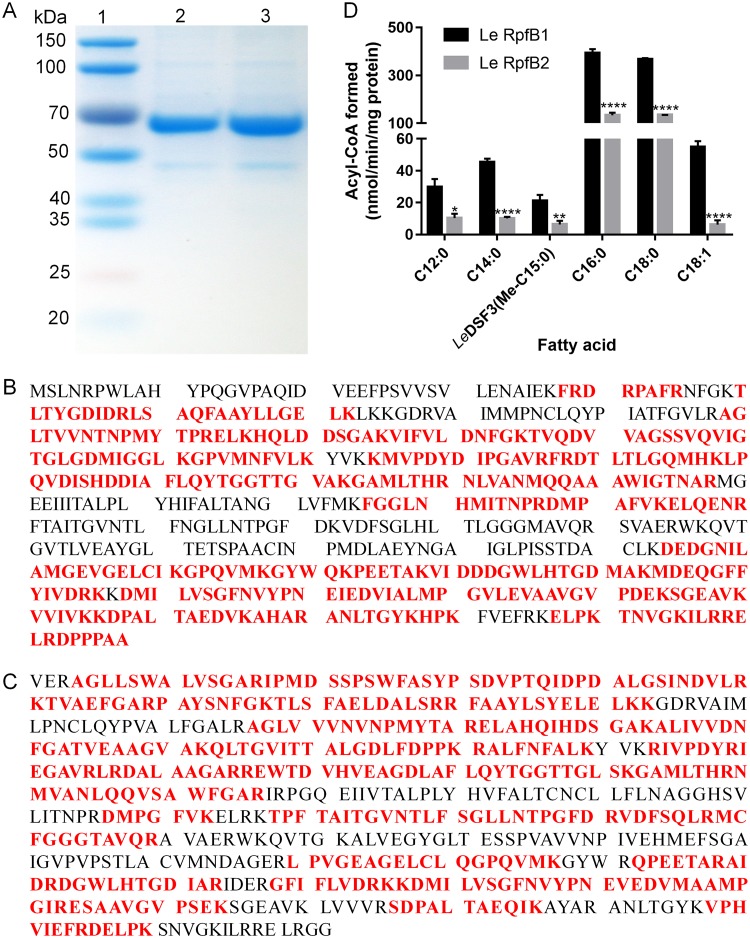
Fatty acyl chain-length specificity of the two *L. enzymogenes* RpfBs. (A) Purification of *L. enzymogenes* RpfB1 and RpfB2 by native nickel-chelation chromatography. Lane 1, molecular mass markers; lane 2, purified RpfB1; lane 3, purified RpfB2. (B) Mass spectrometric identification of *L. enzymogenes* RpfB1. (C) Mass spectrometric identification of *L. enzymogenes* RpfB2. The matching peptides are shown in bold and marked in red. (D) Activities of RpfB1 and RpfB2 using fatty acids with different chain lengths (at 500 μM) as the substrates. Error bars, means ± standard deviations (*n* = 3). *, *P* < 0.05; **, *P* < 0.01; ****, *P* < 0.0001; assessed by one-way ANOVA. All experiments were repeated three times with similar results.

To further characterize the RpfB1- and RpfB2-encoded acyl-CoA ligases, recombinant N-terminal hexahistidine-tagged RpfB1 and RpfB2 were produced. The proteins had a monomeric molecular weight of 61 kDa and were purified by nickel chelation chromatography to obtain preparations that exhibited single bands by SDS-gel electrophoresis (Fig. 3A); the molecular weight and purity were verified by mass analysis (Fig. 3B and C). The fatty acid substrate specificity of RpfB1 and RpfB2 was determined *in vitro*. RpfB1 and RpfB2 exhibited a high level of activity toward C_16:0_ and C_18:0_ substrates, but the acyl-CoA ligase enzymatic activity of RpfB2 was significantly weaker than that of RpfB1. In contrast, the FCL activity of RpfB1 and RpfB2 was very low with *Le*DSF3 fatty acid substrates (Fig. 3D). These results showed that RpfB1 and RpfB2 are long-chain (C_16_ to C_18_) FCLs.

### RpfB1 and RpfB2 were required for the utilization of free fatty acids.

Since RpfB functionally replaced the E. coli FadD β-oxidation protein, we asked whether RpfB could function in *L. enzymogenes* fatty acid utilization. We tested the growth of the Δ*rpfB1*, Δ*rpfB2*, and Δ*rpfB1B2* strains on various fatty acids as sole carbon sources (Table 1). The Δ*rpfB1* and Δ*rpfB2* strains grew well on glucose, sucrose, and fatty acids, but the Δ*rpfB1B2* double-mutant strain completely failed to grow on any fatty acid tested. When the strain was complemented with a *rpfB1* or *rpfB2* plasmid, it had a growth phenotype similar to that of the wild-type *L. enzymogenes*, which grew well on fatty acids C_10:0_ or longer. These results indicated that RpfB1 and RpfB2 are required for the utilization of fatty acids as a sole carbon source and that RpfB1 and RpfB2 are functionally interchangeable in terms of the utilization of fatty acids of *L. enzymogenes*.

**TABLE 1:** Aerobic growth of *L. enzymogenes* strains on fatty acids of various chain lengths^*a*^

C source	Growth result by strain									
	OH11	Δ*rpfB1*	Δ*rpfB1*/*B1*	Δ*rpfB1*/*B2*	Δ*rpfB2*	Δ*rpfB2*/*B1*	Δ*rpfB2*/*B2*	Δ*rpfB1B2*	Δ*rpfB1B2*/*B1*	Δ*rpfB1B2*/*B2*
Glucose	++++	++++	++++	++++	++++	++++	++++	++++	++++	++++
Sucrose	+++	+++	+++	+++	+++	+++	+++	+++	+++	+++
C_4:0_	−	−	−	−	−	−	−	−	−	−
C_6:0_	−	−	−	−	−	−	−	−	−	−
C_8:0_	−	−	−	−	−	−	−	−	−	−
C_10:0_	++	++	++	++	++	++	++	−	−	−
C_12:0_	++	++	++	++	++	++	++	−	−	−
C_14:0_	++	++	++	++	++	++	++	−	+	++
C_16:0_	++	++	++	++	++	++	++	−	+	++
C_18:0_	++	++	++	++	++	++	++	−	+	++
C_18:1_	+++	+++	+++	+++	+++	+++	+++	−	+	++

a *L. enzymogenes* strains were grown at 28°C on M813 modified medium plates supplemented with 0.4% glucose, 0.4% sucrose, or 0.1% fatty acids with Brij58. The fatty acid was butyrate (C_4:0_), hexanoate (C_6:0_), octanoate (C_8:0_), decanoate (C_10:0_), dodecanoate (C_12:0_), tetradecanoate (C_14:0_), hexadecanoic acid (C_16:0_), octadecanoic acid (C_18:0_), or oleate (C_18:1_). Growth was usually obvious after 4–8 days of aerobic growth. Growth was scored “+” if growth after 6 days was obvious or “−” when no colonies were apparent after 8 days of incubation. Aerobic growth (after 4 days) on a given carbon source was given a score of “++++.”

### RpfBs were responsible for *Le*DSF3 production.

**FIG 4:**
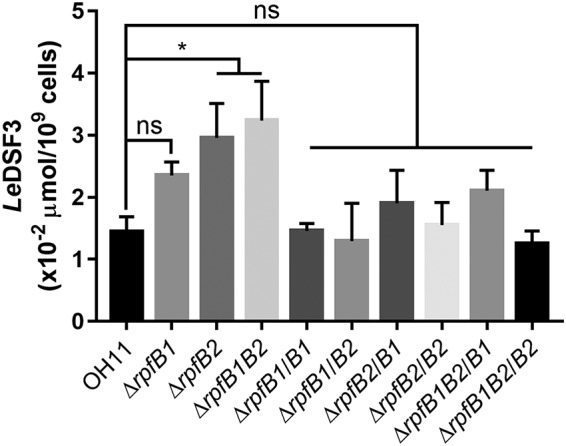
Deletion of *rpfB1* and *rpfB2* affects the synthesis of the *Le*DSF3 signal in *L. enzymogenes*. Error bars, means ± standard deviations (*n* = 3). *, *P* < 0.05; as assessed by one-way ANOVA. All experiments were repeated three times with similar results.

Zhou et al. found that the deletion of *rpfB* resulted in increased DSF production compared with that in X. campestris (12). To investigate whether RpfB modulated *Le*DSF3 production in *L. enzymogenes*, we generated *rpfB1* and *rpfB2* deletion or overexpression strains and measured *Le*DSF3 production in the LB medium supernatant. We found that the amount of *Le*DSF3 signal produced by the Δ*rpfB1*, Δ*rpfB2*, and Δ*rpfB1B2* mutant strains was significantly higher than that produced by the wild-type strain OH11. The *rpfB1*- or *rpfB2*-complemented strains (Δ*rpfB1/B1*, Δ*rpfB1/B2*, Δ*rpfB2/B1*, Δ*rpfB2/B2*, Δ*rpfB1B2/B1*, and Δ*rpfB1B2/B2*) yielded *Le*DSF3 signals at the level observed with the wild-type strain OH11 (Fig. 4). These findings indicated that RpfB1 and RpfB2 are responsible for affecting the synthesis of the *Le*DSF3 signal in *L. enzymogenes*.

### *L. enzymogenes* Clp directly positively regulated RpfB1 and RpfB2 expression.

**FIG 5:**
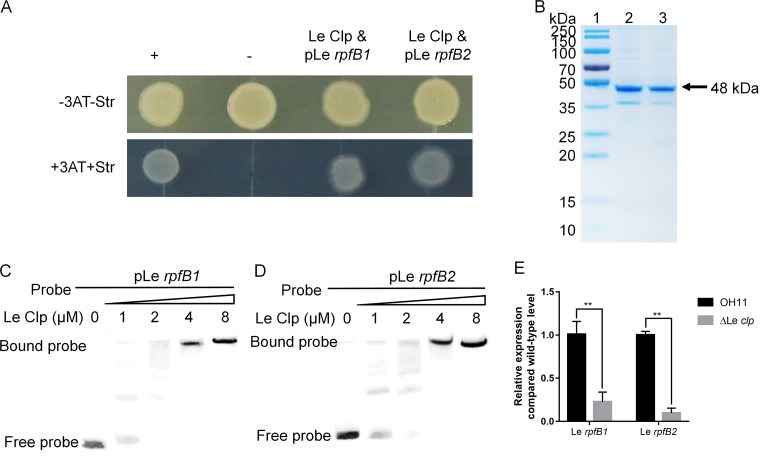
*L. enzymogenes* Clp directly bound the promoters of *rpfB1* and *rpfB2*. (A) Direct physical interaction between Clp and the *rpfB1* and *rpfB2* promoter regions was detected in E. coli. Experiments were performed according to the procedures described in the Materials and Methods. +, cotransformant containing pBX-R2031 and pTRG-R3133, used as a positive control; −, cotransformant containing pBXcmT and the empty pTRG, used as a negative control; Clp and pLe *rpfB1*, cotransformant harboring pTRG-clp and pBXcmT-pLe *rpfB1*. Clp and pLe *rpfB2*, cotransformant harboring pTRG-Clp and pBXcmT-pLe *rpfB2*. −3AT-Str, plate with no selective medium (3AT, 3-amino-1,2,4-triazole and Str, streptomycin); +3AT+Str, plate with M9-based selective medium. (B) The purified protein was analyzed by 12% SDS-PAGE. Lane 1, molecular mass markers; lanes 2 to 3, Clp protein. (C and D) Gel shift assay showing that Clp directly regulates *rpfB1* or *rpfB2*. Clp (0, 1, 2, 4, or 8 μM) was added to reaction mixtures containing 50 ng of probe DNA, and the reaction mixtures were separated on polyacrylamide gels. (E) Relative expression of *rpfB1* and *rpfB2*, as determined by qRT-PCR. All experiments were repeated three times with similar results.

Previously, Zhou et al. showed that Clp binds to the upstream region of X. campestris *rpfB* (12). Based on the DNA consensus sequence 5′-ATGC-N6-GCAT-3′, which was identified for X. campestris Clp (12), we identified the following two potential binding sites of *L. enzymogenes* Clp in the upstream region of *rpfB1* and *rpfB2*: 5′-ATGC-N22-CGAT-3′ and 5′-ATCG-N22-GCAT-3′ (see Fig. S2 in the supplemental material). We, therefore, hypothesized that *L. enzymogenes* Clp mediates the effects of *rpfB1* and *rpfB2* on HSAF biosynthesis. To test this hypothesis, we performed an E. coli-based one-hybrid assay and found that Clp could directly bind to the promoters of *L. enzymogenes rpfB1* and *rpfB2* (Fig. 5A). To further verify this binding, an electrophoretic mobility shift assay (EMSA) was performed. The putative promoter DNA fragments covering 451 bp or 486 bp upstream of the *L. enzymogenes rpfB1* or *rpfB2* translational start site, namely, pLe *rpfB1* or pLe *rpfB2*, respectively, were cloned and analyzed using EMSA. The addition of purified glutathione *S*-transferase (GST)-Clp protein (Fig. 5B) at concentrations ranging from 0 μM to 8 μM to the reaction mixtures (20 μl, 28°C, 25 min) caused a shift in the mobility of the pLe *rpfB1* or pLe *rpfB2* DNA fragment, which suggests that Clp directly binds to the promoter regions of *rpfB1* and *rpfB2* (Fig. 5C and D). Reverse transcription-quantitative PCR (qRT-PCR) analysis showed that the expression of *L. enzymogenes rpfB1* or *rpfB2* in the Δ*Le clp* mutant strain resulted in decreased RpfB1 and RpfB2 expression (Fig. 5E). Thus, the above results collectively suggest that *L. enzymogenes* Clp directly binds to the promoters of *rpfB1* and *rpfB2* to promote expression.

### Deletion of *rpfB1* and *rpfB2* resulted in increased HSAF production.

**FIG 6:**
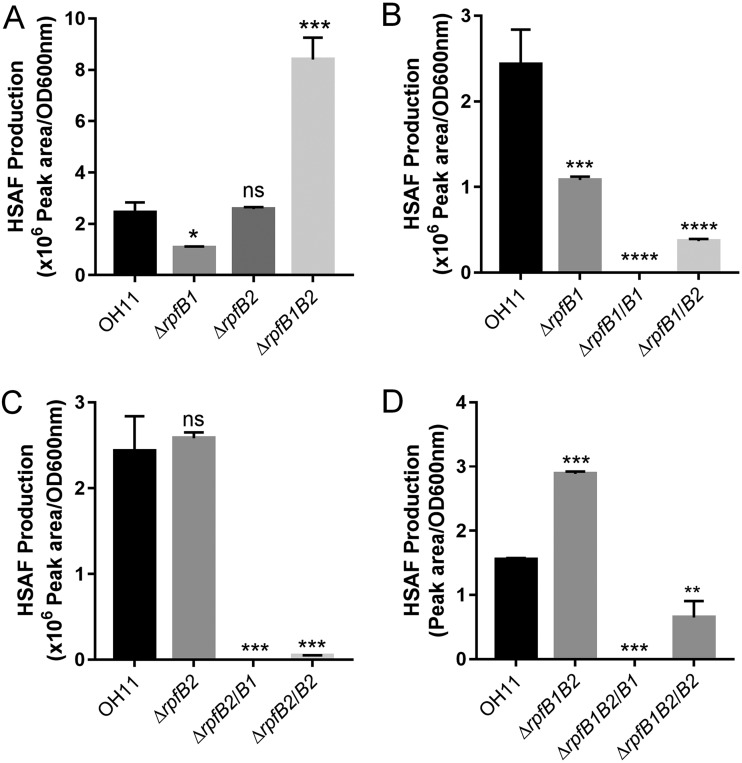
Deletion of *rpfB1B2* resulted in increased HSAF production, and HSAF production in the *rpfB1*- and *rpfB2*-complemented strains was completely suppressed. (A) Quantification of HSAF in the *rpfB* mutant strains. (B) Quantification of HSAF in the *rpfB1* mutant strains complemented with the *rpfB1* gene. (C) Quantification of HSAF in the *rpfB2* mutant strains complemented with the *rpfB2* gene. (D) Quantification of HSAF in the *rpfB1B2* double-mutant strains complemented with the *rpfB1* or *rpfB2* gene. Error bars, means ± standard deviations (*n* = 3). *, *P* < 0.05; **, *P* < 0.01; ***, *P* < 0.001; ****, *P* < 0.0001; assessed by one-way ANOVA. All experiments were repeated three times with similar results.

*L. enzymogenes* is known for its synthesis of the antifungal antibiotic HSAF (20). To identify the physiological functions of *L. enzymogenes* RpfB1 and RpfB2 in HSAF production, the knockout strains Δ*rpfB1* and Δ*rpfB2* and the double-mutant strain Δ*rpfB1B2*, in which the *rpfB1* (Le4724) and *rpfB2* (Le2453) genes were deleted, were constructed by a two-step homologous recombination approach. We quantified HSAF production in the Δ*rpfB1* and Δ*rpfB2* single mutants and in the double-mutant strain Δ*rpfB1B2* by use of a high-performance liquid chromatograph (HPLC). We found that Δ*rpfB1* exhibited decreased HSAF levels and that the deletion of *rpfB2* did not affect HSAF levels. However, Δ*rpfB1B2* exhibited a significant increase in HSAF levels compared with the wild-type levels (Fig. 6A). To determine the role of RpfB1 and RpfB2 in the regulation of HSAF biosynthesis, we complemented the Δ*rpfB1*, Δ*rpfB2*, and Δ*rpfB1B2* mutants with plasmid-borne *rpfB1* and *rpfB2*. HSAF production in the *rpfB1*-complemented strains (Δ*rpfB1/B1*, Δ*rpfB2/B1*, and Δ*rpfB1B2/B1*) was completely suppressed, and in the *rpfB2*-complemented strains (Δ*rpfB1/B2*, Δ*rpfB2/B2*, and Δ*rpfB1B2/B2*), HSAF production was significantly decreased (Fig. 6B, C, and D). Importantly, the *rpfB1*, *rpfB2*, and *rpfB1B2* mutations did not impair bacterial growth (see Fig. S3 in the supplemental material), implying that *L. enzymogenes* RpfB1 and RpfB2 play a specific role in regulating HSAF production. In previous work, Glu-361 was found to be critical for the FCL activity of RpfB1 and RpfB2 (Fig. 2); therefore, we expected the *rpfB1* E361A and *rpfB2* E361A mutants to not reduce HSAF production in the Δ*rpfB1*, Δ*rpfB2*, and Δ*rpfB1B2* mutant strains. To test this prediction, we complemented the Δ*rpfB1*, Δ*rpfB2*, and Δ*rpfB1B2* mutants with the plasmid-borne *rpfB1* E361A and *rpfB2* E361A mutants. We found that mutation of Glu-361 to Ala-361 did not significantly reduce HSAF production compared with that in the Δ*rpfB1*, Δ*rpfB2*, and Δ*rpfB1B2* mutant strains (see Fig. S4 in the supplemental material).

### Intracellular free fatty acids were critical for HSAF production.

**FIG 7:**
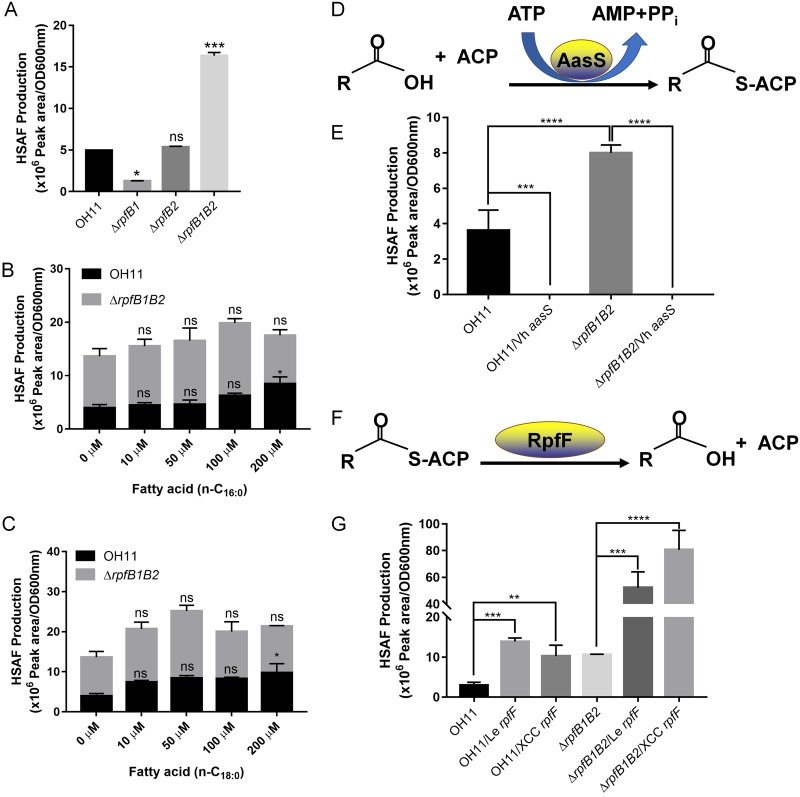
Intracellular free fatty acids were required for HSAF biosynthesis. (A) Quantification of HSAF produced by the Δ*rpfB1*, Δ*rpfB2*, and Δ*rpfB1B2* mutant strains grown in 10% TSB medium without free fatty acids. (B) Quantification of HSAF produced by the *rpfB* mutant strains grown in 10% TSB medium supplemented with hexadecanoic acid (n-C_16:0_). (C) Quantification of HSAF produced by the *rpfB* mutant strains grown in 10% TSB medium supplemented with octadecanoic acid (n-C_18:0_). (D) Chemical equation of the acyl-ACP synthetase reaction. (E) Quantification of HSAF produced by the wild-type OH11 and *rpfB1B2* double-mutant strains complemented with the V. harveyi *aasS* gene. (F) Chemical equation of the acyl-ACP thioesterase reaction. (G) Quantification of HSAF produced by the wild-type OH11 and the *rpfB1B2* double-mutant strains complemented with the *L. enzymogenes rpfF* or X. campestris *rpfF* gene. Error bars, means ± standard deviations (*n* = 3). *, *P* < 0.05; **, *P* < 0.01; ***, *P* < 0.001; ****, *P* < 0.0001; assessed by one-way ANOVA. All experiments were repeated three times with similar results.

The above studies also found that HSAF production in the *rpfB1*- and *rpfB2*-complemented strains was completely suppressed compared with that in the Δ*rpfB1*, Δ*rpfB2*, and Δ*rpfB1B2* mutants (Fig. 6B, C, and D). RpfB is an acyl-CoA ligase that catalyzes the synthesis of acyl-CoA from free fatty acids and coenzyme A (CoA) (Fig. 2 and 3). We hypothesized that the in-frame deletion of *rpfB1* and *rpfB2* affected HSAF production due to the influence of the free fatty acid content. To test this hypothesis, we first determined the effect of 10% Trypticase soy broth (TSB) medium without free fatty acids on the synthesis of HSAF in *L. enzymogenes*. We found that extracellular free fatty acids do not affect HSAF synthesis in *L. enzymogenes* (Fig. 7A). To further investigate the function of extracellular free fatty acids in HSAF production, we examined HSAF synthesis in *L. enzymogenes* grown in 10% TSB medium supplemented with free fatty acids (n-C_16:0_ or n-C_18:0_). As shown in Fig. 7B and C, these conditions weakly promoted HSAF production by wild-type OH11, but none of these conditions significantly promoted HSAF production by the Δ*rpfB1B2* mutant strain. These results indicated that extracellular free fatty acids were not key for HSAF production in *L. enzymogenes*. To further determine the importance of intracellular free fatty acids for HSAF synthesis in *L. enzymogenes*, we overexpressed a Vibrio harveyi acyl-acyl carrier protein (ACP) ligase-encoding gene (Vh *aasS*) that catalyzes the synthesis of acyl-ACP from free fatty acids and ACP (Fig. 7D) (21) in both the wild-type OH11 and Δ*rpfB1B2* mutant backgrounds, and the resulting decreased levels of intracellular free fatty acids can completely suppress HSAF production (Fig. 7E). To ultimately determine whether RpfBs affect intracellular free fatty acid metabolism to regulate the synthesis of HSAF, we overexpressed *L. enzymogenes* and X. campestris acyl-ACP thioesterases (*rpfF* of both species), which cleave acyl-ACP thioester bonds to yield free fatty acids plus holo-ACP (Fig. 7F) (13, 22), in both the wild-type OH11 and Δ*rpfB1B2* mutant strains, and the resulting increase in intracellular free fatty acids significantly increased HSAF production (Fig. 7G). We quantified the cellular fatty acid content and found that Δ*rpfB1* exhibited decreased cellular fatty acid levels and that the deletion of *rpfB2* did not affect the cellular fatty acid content. However, Δ*rpfB1B2* exhibited a significant increase in cellular fatty acid content compared with that of the wild type. This result is consistent with HSAF production (see Fig. S5 in the supplemental material). These findings suggested that RpfBs affect intracellular free fatty acid metabolism to block HSAF biosynthesis.

## DISCUSSION

Previous studies have indicated that FCL is widespread in a variety of bacterial species and that RpfB encodes an acyl-CoA ligase (15, 17, 23, 24). Recent studies have shown that RpfB represents a naturally occurring signal turnover system that targets DSF-family QS signals in X. campestris (12). *Le*DSF3-mediated QS plays critical roles in regulating the biosynthesis of HSAF in *L. enzymogenes* (10, 14). However, the mechanism through which RpfB coordinates the synthesis of HSAF remains unknown. In this study, we investigated the RpfB-dependent regulation of the biosynthesis of the antibiotic HSAF in *L. enzymogenes*.

Unlike many other bacteria, *L. enzymogenes* has two FCLs for long-chain fatty acid activation (Fig. 1). The present study demonstrated that RpfB1 and RpfB2 affect the synthesis of the *Le*DSF3 signal in *L. enzymogenes* (Fig. 4). The FCLs RpfB1 and RpfB2 exhibited a low level of activity toward *Le*DSF3 fatty acid substrates (Fig. 3D). These results are indicative of the presence of a RpfB-dependent signal turnover system in *L. enzymogenes*.

Our data show that the in-frame deletion of the *rpfB1* or *rpfB2* coding sequence does not significantly affect HSAF production (Fig. 6). However, surprisingly, the deletion of both the *rpfB1* and *rpfB2* genes resulted in a significant increase in HSAF production (Fig. 6A and D), and the overexpression of *rpfB1* or *rpfB2* in the wild-type OH11 strain and the Δ*rpfB1*, Δ*rpfB2*, and Δ*rpfB1B2* mutant strains completely suppressed HSAF production (Fig. 6B, C, and D). RpfB1 and RpfB2 have been shown to exhibit long-chain (n-C_16:0_ and n-C_18:0_) FCL activity and harbor a key catalytic glutamine residue (Glu-361) that is required for the FCL activity of RpfB1 and RpfB2 (Fig. 2). This activity is different from the catalytic activity of E. coli FadD and X. campestris RpfB with n-C_12:0_ and n-C_14:0_ free fatty acids as the substrates (15, 17). Apparently, RpfB1 and RpfB2 use their FCL activity to affect free fatty acid metabolism and, thus, block HSAF biosynthesis in *L. enzymogenes*. Therefore, we tested the effect of inactive RpfB1 and RpfB2 on HSAF synthesis; RpfB1 and RpfB2 with point mutations of the E-361 residue were expressed in the wild-type OH11 strain and in the Δ*rpfB1*, Δ*rpfB2*, and Δ*rpfB1B2* mutant strains. Our analysis revealed that residue Glu-361 in RpfB1 and RpfB2 was essential for blocking HSAF biosynthesis (see Fig. S4 in the supplemental material). Importantly, we found that RpfB2 is outside the Rpf gene cluster and plays a key role in HSAF production, which has not been reported in other DSF/Rpf-producing bacteria (11, 12, 15).

The *aasS* gene of V. harveyi B392 is a unique gene that encodes acyl-ACP synthetase, which catalyzes the synthesis of acyl-ACP from free fatty acids and ACP (21). Thus, to verify the importance of intracellular free fatty acids for HSAF synthesis, we overexpressed V. harveyi acyl-ACP synthetase (AasS) and successfully showed that this protein could recapture intracellular fatty acids in *L. enzymogenes* and completely suppress HSAF production (Fig. 7E). In addition, we found that extracellular free fatty acids do not affect HSAF production in *L. enzymogenes* (Fig. 7A, B, and C). Expression of acyl-ACP thioesterase could increase intracellular free fatty acid production (22). Thus, we overexpressed *L. enzymogenes* and X. campestris acyl-ACP thioesterases (*rpfF* of both species), which increased HSAF production in *L. enzymogenes* (Fig. 7G). These data clearly show that increasing or decreasing the levels of intracellular free fatty acids can significantly increase or decrease the synthesis of HSAF, suggesting that RpfBs affect intracellular free fatty acid metabolism to block HSAF biosynthesis via FCL activity in *L. enzymogenes*. However, the detailed mechanism underlying the effect of intracellular free fatty acids on HSAF production remains to be studied. Li et al. speculated that the synthetic precursor of HSAF may be a polyene-hexaketide fatty acid (8). Therefore, we speculated that compared with RpfB1, RpfB2 has higher enzymatic activity when using polyene-hexaketide fatty acid as a substrate. However, we cannot obtain the polyene-hexaketide fatty acid, so the ultimate mechanism still needs to be studied in the future. The present study demonstrated that free fatty acids are usually converted to acyl-ACP or acyl-CoA, which are involved in cellular metabolism in bacteria (25–28). However, our study found that acyl-ACP and acyl-CoA are not required for HSAF synthesis in *L. enzymogenes*. Therefore, we speculate that *L. enzymogenes* can synthesize HSAF using intracellular free fatty acids as potential substrates. Further clarification of these possible mechanisms will help elucidate the mechanism underlying the use of intracellular free fatty acids as potential substrates for HSAF synthesis.

Clp has been well studied as a cyclic di-GMP-responsive transcriptional regulator that plays essential roles in virulence factor production and in the DSF signaling network (29, 30). Previous findings have demonstrated that Clp is the major regulator of HSAF biosynthesis gene expression in *L. enzymogenes* (20, 31). The present study revealed that Clp acts as a transcriptional activator to directly positively regulate RpfB1 and RpfB2 expression (Fig. 5), which is different from observations in X. campestris (12). We can only speculate that *L. enzymogenes* Clp not only directly regulates the biosynthesis of HSAF but also indirectly regulates other factors, such as RpfB, to affect HSAF production.

Overall, *L. enzymogenes* Clp positively regulated RpfB1 and RpfB2 expression via direct binding to their promoters. RpfBs could affect intracellular free fatty acid metabolism to block the biosynthesis of HSAF via FCL activity in *L. enzymogenes*.

## MATERIALS AND METHODS

### Materials.

The antibiotics used in this study were obtained from Sigma-Aldrich (St. Louis, MO, USA). TaKaRa Biotechnology Co. (Dalian, Liaoning, China) provided the molecular biology reagents, and Novagen (Madison, WI, USA) provided the pET vectors. Ni-agarose columns were obtained from Sigma-Aldrich, and Bio-Rad (Hercules, CA, USA) provided the Quick Start Bradford dye reagent. All other reagents used in this study were of the highest purity available. Genscript (Nanjing, Jiangsu, China) synthesized the oligonucleotide primers.

### Bacterial strains, plasmids, and growth conditions.

The strains and plasmids used in this study are shown in Table 2. E. coli strains were grown in Luria-Bertani medium (10 g/liter tryptone, 5 g/liter yeast extract, and 10 g/liter NaCl [pH 7.0]) at 37°C. *L. enzymogenes* strains were grown at 28°C in Luria-Bertani medium and 10% TSB. M813 modified medium (4 g glucose, 3 g K_2_HPO_4_, 1.2 g NaH_2_PO_4_, 1 g NH_4_Cl, 0.3 g MgSO_4_, 0.15 g KCl, 10 mg CaCl_2_, and 2.8 mg FeSO_4_·7H_2_O, per liter) was used for the growth of *L. enzymogenes* OH11 (32). For the preparation of culture medium, tryptone, peptone, beef extract, and yeast extract were purchased from Sangon Biotech (Shanghai, China). When required, antibiotics were added (100 μg/ml sodium ampicillin, 30 μg/ml kanamycin sulfate, and 50 μg/ml gentamicin) to the E. coli or *L. enzymogenes* cultures. The bacterial growth in liquid medium was determined by measuring the optical density at 600 nm (OD_600_) using a Bioscreen-C automated growth curves analysis system (Oy Growth Curves, Helsinki, Finland).

**TABLE 2:** Bacterial strains and plasmids used in this study

Bacterial strain or plasmid	Relevant characteristics^*a*^	Reference
Strains		
E. coli		
BL21(DE3)	F^−^ *dcm ompT hsdS*(r_B_^−^m_B_^−^) *gal* (λDE3)	Lab collection
DH5α	F^−^ *deoR endA1 gyrA96 hsdR17*(r_K_^−^m_K_^+^) *recA1 relA1 supE44 thi-1* Δ(*lacZYA-argF*)*U169*(φ80*lacZ*ΔM15)	Lab collection
JW1794	BW25113 Δ*fadD*::Kan^R^	19
XL1-Blue MRF′ kan	Δ(*mcrA*)183, Δ(*mcrCB-hsdSMR-mrr*)173, *endA1*, *supE44*, *thi-1*, *recA1 gyrA96*, *relA1*, *lac*, [F′ *proAB lacI*^q^*Z*ΔM15 Tn*5* (Km^R^)]	42
*L. enzymogenes*		
OH11	Kan^R^, wild-type strain	Lab stock
Δ*rpfB1*	Kan^R^, the *L. enzymogenes rpfB1* in-frame deletion mutant of strain OH11	This study
Δ*rpfB2*	Kan^R^, the *L. enzymogenes rpfB2* in-frame deletion mutant of strain OH11	This study
Δ*rpfB1B2*	Kan^R^, the *L. enzymogenes rpfB1* and *rpfB2* in-frame deletion mutant of strain OH11	This study
Δ*rpfB1*/*B1*	Kan^R^, Gm^R^, the *rpfB1* in-frame deletion mutant harboring the *rpfB1* expression plasmid pBBR1-*rpfB1*	This study
Δ*rpfB1*/*B2*	Kan^R^, Gm^R^, the *rpfB1* in-frame deletion mutant harboring the *rpfB2* expression plasmid pBBR1-*rpfB2*	This study
Δ*rpfB2*/*B1*	Kan^R^, Gm^R^, the *rpfB2* in-frame deletion mutant harboring the *rpfB1* expression plasmid pBBR1-*rpfB1*	This study
Δ*rpfB2*/*B2*	Kan^R^, Gm^R^, the *rpfB2* in-frame deletion mutant harboring the *rpfB2* expression plasmid pBBR1-*rpfB2*	This study
Δ*rpfB1B2*/*B1*	Kan^R^, Gm^R^, the *rpfB1* and *rpfB2* double-mutant strain harboring the *rpfB1* expression plasmid pBBR1-*rpfB1*	This study
Δ*rpfB1B2*/*B2*	Kan^R^, Gm^R^, the *rpfB1* and *rpfB2* double-mutant strain harboring the *rpfB2* expression plasmid pBBR1-*rpfB2*	This study
Δ*rpfB1*/*B1* E361A	Kan^R^, Gm^R^, the *rpfB1* in-frame deletion mutant harboring the *rpfB1* E361A expression plasmid pBBR1-*rpfB1* E361A	This study
Δ*rpfB2*/*B2* E361A	Kan^R^, Gm^R^, the *rpfB2* in-frame deletion mutant harboring the *rpfB2* E361A expression plasmid pBBR1-*rpfB2* E361A	This study
Δ*rpfB1B2*/*B1* E361A	Kan^R^, Gm^R^, the *rpfB1* and *rpfB2* double-mutant strain harboring the *rpfB1* E361A expression plasmid pBBR1-*rpfB1* E361A	This study
Δ*rpfB1B2*/*B2* E361A	Kan^R^, Gm^R^, the *rpfB1* and *rpfB2* double-mutant strain harboring the *rpfB2* E361A expression plasmid pBBR1-*rpfB2* E361A	This study
OH11/Vh *aasS*	Kan^R^, Gm^R^, the wild-type strain OH11 harboring the V. harveyi *aasS* expression plasmid pBBR1-Vh *aasS*	This study
Δ*rpfB1B2*/Vh *aasS*	Kan^R^, Gm^R^, the *rpfB1* and *rpfB2* double-mutant strain harboring the V. harveyi *aasS* expression plasmid pBBR1-Vh *aasS*	This study
OH11/Le *rpfF*	Kan^R^, Gm^R^, the wild-type strain OH11 harboring the *L. enzymogenes rpfF* expression plasmid pBBR1-Le *rpfF*	This study
OH11/XCC *rpfF*	Kan^R^, Gm^R^, the wild-type strain OH11 harboring the X. campestris *rpfF* expression plasmid pBBR1-XCC *rpfF*	This study
Δ*rpfB1B2*/Le *rpfF*	Kan^R^, Gm^R^, the *rpfB1* and *rpfB2* double-mutant strain harboring the *L. enzymogenes rpfF* expression plasmid pBBR1-Le *rpfF*	This study
Δ*rpfB1B2*/XCC *rpfF*	Kan^R^, Gm^R^, the *rpfB1* and *rpfB2* double-mutant strain harboring the *L. enzymogenes rpfF* expression plasmid pBBR1-Le *rpfF*	This study
ΔLe *clp*	Kan^R^, the *L. enzymogenes clp* in-frame deletion mutant of strain OH11	40
Plasmids		
pET28b	Km^R^, T7 promoter-based expression vector	Lab collection
pBAD24M	Amp^R^; P_BAD_ promoter-based expression vector	34
pEX18GM	Gm^R^, *sacB*-based gene replacement vector	37
pBBR1MCS5	Gm^R^, broad host range cloning vector	38
pET-*rpfB1*	Km^R^, *L. enzymogenes rpfB1* in pET-28b	This study
pET-*rpfB2*	Km^R^, *L. enzymogenes rpfB2* in pET-28b	This study
pBAD24M-*rpfB1*	Amp^R^, *L. enzymogenes rpfB1* in pBAD24M	This study
pBAD24M-*rpfB2*	Amp^R^, *L. enzymogenes rpfB2* in pBAD24M	This study
pBAD24M-*rpfB1* Y213A	Amp^R^, RpfB1 Y213A in pBAD24M-*rpfB1*	This study
pBAD24M-*rpfB1* T214A	Amp^R^, RpfB1 T214A in pBAD24M-*rpfB1*	This study
pBAD24M-*rpfB1* G216A	Amp^R^, RpfB1 G216A in pBAD24M-*rpfB1*	This study
pBAD24M-*rpfB1* T217A	Amp^R^, RpfB1 T217A in pBAD24M-*rpfB1*	This study
pBAD24M-*rpfB1* G219A	Amp^R^, RpfB1 G219A in pBAD24M-*rpfB1*	This study
pBAD24M-*rpfB1* E361A	Amp^R^, RpfB1 E361A in pBAD24M-*rpfB1*	This study
pBAD24M-*rpfB2* Y213A	Amp^R^, RpfB2 Y213A in pBAD24M-*rpfB2*	This study
pBAD24M-*rpfB2* T214A	Amp^R^, RpfB2 T214A in pBAD24M-*rpfB2*	This study
pBAD24M-*rpfB2* G216A	Amp^R^, RpfB2 G216A in pBAD24M-*rpfB2*	This study
pBAD24M-*rpfB2* T217A	Amp^R^, RpfB2 T217A in pBAD24M-*rpfB2*	This study
pBAD24M-*rpfB2* G219A	Amp^R^, RpfB2 G219A in pBAD24M-*rpfB2*	This study
pBAD24M-*rpfB2* E361A	Amp^R^, RpfB2 E361A in pBAD24M-*rpfB2*	This study
pBBR1-*rpfB1*	Gm^R^, *L. enzymogenes rpfB1* in pBBR1MCS5	This study
pBBR1-*rpfB2*	Gm^R^, *L. enzymogenes rpfB2* in pBBR1MCS5	This study
pBBR1-*rpfB1* E361A	Gm^R^, RpfB1 E361A in pBBR1-*rpfB1*	This study
pBBR1-*rpfB2* E361A	Gm^R^, RpfB2 E361A in pBBR1-*rpfB2*	This study
pBBR1-Vh *aasS*	Gm^R^, V. harveyi *aasS* in pBBR1MCS5	This study
pBBR1-Le *rpfF*	Gm^R^, *L. enzymogenes rpfF* in pBBR1MCS5	This study
pBBR1-XCC *rpfF*	Gm^R^, X. campestris *rpfF* in pBBR1MCS5	This study
pEX18-Δ*rpfB1*	Gm^R^, *L. enzymogenes rpfB1* in-frame deletion fragment inserted into pEX18GM vector between HindIII/XbaI sites	This study
pEX18-Δ*rpfB2*	Gm^R^, *L. enzymogenes rpfB2* in-frame deletion fragment inserted into pEX18GM vector between HindIII/XbaI sites	This study
pGEX-6p-1	Amp^R^, vector for protein expression	45
pGEX-6p-1-Clp	Amp^R^, pGEX-6p-1 with the coding region of Clp	40
pTRG	Tet^R^, plasmid used for protein expression in bacterial one-hybrid assay	42
pTRG-Clp	Tet^R^, pTRG with the coding region of Clp	45
pBXcmT	Cm^R^, plasmid used for DNA cloning in bacterial one-hybridization assay	40
pBXcmT-pLe *rpfB1*	Cm^R^, pBXcmT with the *L. enzymogenes rpfB1* promoter region	This study
pBXcmT-pLe *rpfB2*	Cm^R^, pBXcmT with the *L. enzymogenes rpfB2* promoter region	This study

a Km^R^, kanamycin resistance; Gm^R^, gentamicin resistance; Amp^R^, ampicillin resistance; Cm^R^, chloramphenicol resistance.

### Complementation of an E. coli Δ*fadD* strain.

An E. coli Δ*fadD* strain complementation assay was performed as previously described (33). An *fadD* mutant strain of E. coli (JW1794) was used for the complementation studies (19). *L. enzymogenes rpfB* genes were amplified from the genomic DNA of the wild-type OH11 strain using the primers listed in Table 3, which contained NdeI and HindIII sites. PCR fragments were purified and cloned into pBAD24M (34). The *rpfB* sequences were verified by sequencing. The two recombinant vectors were introduced into the E. coli strain JW1794 for complementation. An empty vector was also transformed into JW1794 and used as a negative control in the complementation experiments. The E. coli *fadD* gene was also employed as a control for the complementation study. These strains were inoculated into M9 minimal medium supplemented with 0.1% fatty acids with Brij58 (0.02% arabinose was added if required), and the complementation results were determined after the cells were incubated for 72 h at 37°C.

**TABLE 3:** Sequences of the PCR primers used in this work

Primer name by use	Primer sequence (5′ to 3′)^*a*^	Digestion site
For deletion		
*rpfB1* HindIII	ATCCAAGCTTCATCCTGCAACTGGGTCATC	HindIII
*rpfB1* up1	TGACGATCACCACCTTGACCTAGGTCAGTGTCTTGCCGAA	
*rpfB1* down1	TTCGGCAAGACACTGACCTAGGTCAAGGTGGTGATCGTCA	
*rpfB1* XbaI	CTAGTCTAGAATGTCTTCCATCAGCGGTGT	XbaI
*rpfB2* HindIII	ACCCAAGCTTGCAGACCGAACTCAACTTCA	HindIII
*rpfB2* up1	CACCTTGTACCCCGTGAGATGGTCTTGCCGAAATTGCTGT	
*rpfB2* down1	ACAGCAATTTCGGCAAGACCATCTCACGGGGTACAAGGTG	
*rpfB2* XbaI	CTAGTCTAGACCATCTTGATCCTGCCGTTG	XbaI
For *in trans* expression		
pBBR1-*rpfB1*-F	ATGGGGTACCCATCCTGCAACTGGGTCATC	KpnI
pBBR1-*rpfB1*-R	ACCCAAGCTTTTGCCTGTTTCGCTGCAGGA	HindIII
pBBR1-*rpfB2*-F	ATGGGGTACCGCAGAAGACCATGTACGGC	KpnI
pBBR1-*rpfB2*-R	ACCCAAGCTTCCACCAAGACAACAAAGGCA	HindIII
24M-*rpfB1* F	GAATTCCATATGATGAGTTTGAACCGTCCGTG	NdeI
24M -*rpfB1* R	ACCCAAGCTTTTGCCTGTTTCGCTGCAGGA	HindIII
24M -*rpfB2* F	GAATTCCATATGGTGGAGCGCGCCGGCTTGCT	NdeI
24M -*rpfB2* R	ACCCAAGCTTCCACCAAGACAACAAAGGCA	HindIII
Le *rpfB1* Y213A F	ACATCGCGTTCCTGCAGGCAACCGGCGGCACCACCGGC	
Le *rpfB1* Y213A R	GCCGGTGGTGCCGCCGGTTGCCTGCAGGAACGCGATGT	
Le *rpfB1* T214A F	TCGCGTTCCTGCAGTACGCAGGCGGCACCACCGGCGTG	
Le *rpfB1* T214A R	CACGCCGGTGGTGCCGCCTGCGTACTGCAGGAACGCGA	
Le *rpfB1* G216A F	TCCTGCAGTACACCGGCGCAACCACCGGCGTGGCCAAG	
Le *rpfB1* G216A R	CTTGGCCACGCCGGTGGTTGCGCCGGTGTACTGCAGGA	
Le *rpfB1* T217A F	TGCAGTACACCGGCGGCGCAACCGGCGTGGCCAAGGG	
Le *rpfB1* T217A R	CCCTTGGCCACGCCGGTTGCGCCGCCGGTGTACTGCA	
Le *rpfB1* G219A F	ACACCGGCGGCACCACCGCAGTGGCCAAGGGCGCCATG	
Le *rpfB1* G219A F	ACACCGGCGGCACCACCGCAGTGGCCAAGGGCGCCATG	
Le *rpfB1* E361A F	AGGCCTACGGCCTGACCGCAACCTCGCCGGCGGCGTGC	
Le *rpfB1* E361A R	GCACGCCGCCGGCGAGGTTGCGGTCAGGCCGTAGGCCT	
Le *rpfB2* Y213A F	ACCTCGCGTTCCTGCAGGCAACCGGCGGCACCACCGGC	
Le *rpfB2* Y213A R	GCCGGTGGTGCCGCCGGTTGCCTGCAGGAACGCGAGGT	
Le *rpfB2* T214A F	TCGCGTTCCTGCAGTACGCAGGCGGCACCACCGGCCT	
Le *rpfB2* T214A R	AGGCCGGTGGTGCCGCCTGCGTACTGCAGGAACGCGA	
Le *rpfB2* G216A F	TCCTGCAGTACACCGGCGCAACCACCGGCCTGTCCAAG	
Le *rpfB2* G216A R	CTTGGACAGGCCGGTGGTTGCGCCGGTGTACTGCAGGA	
Le *rpfB2* T217A F	TGCAGTACACCGGCGGCGCAACCGGCCTGTCCAAGGGC	
Le *rpfB2* T217A R	GCCCTTGGACAGGCCGGTTGCGCCGCCGGTGTACTGCA	
Le *rpfB2* G219A F	ACACCGGCGGCACCACCGCACTGTCCAAGGGCGCGATG	
Le *rpfB2* G219A R	CATCGCGCCCTTGGACAGTGCGGTGGTGCCGCCGGTGT	
Le *rpfB2* E361A F	AAGGCTACGGCCTGACCGCAAGTTCGCCGGTGGCGGTG	
Le *rpfB2* E361A R	CACCGCCACCGGCGAACTTGCGGTCAGGCCGTAGCCTT	
For protein expression		
28b-*rpfB1* P1	GAATTCCATATGATGAGTTTGAACCGTCCGTG	NdeI
28b-*rpfB1* P2	ACCCAAGCTTTTGCCTGTTTCGCTGCAGGA	HindIII
28b-*rpfB2* P1	GAATTCCATATGGTGGAGCGCGCCGGCTTGCT	NdeI
28b-*rpfB2* P2	ACCCAAGCTTCCACCAAGACAACAAAGGCA	HindIII
For bacterial one-hybrid assays and EMSA		
p*rpfB1* P1	CCGCTCGAGCATCCTGCAACTGGGTCATC	XhoI
p*rpfB1* P2	TGCTCTAGACACGGACGGTTCAAACTCAT	XbaI
p*rpfB2* P1	CCGCTCGAGGCAGAAGACCATGTACGGC	XhoI
p*rpfB2* P2	TGCTCTAGAAGCAAGCCGGCGCGCTCCAC	XbaI
For RT-PCR		
RT-*rpfB1*-F	AGAAGATGGTGCCCGACTAC	
RT-*rpfB1*-R	GATGATCTCTTCGCCCATGC	
RT-*rpfB2*-F	GTCAATCCGATGTACACCGC	
RT-*rpfB2*-R	GACGTACTTGAGCGCGAAAT	
RT-16S rRNA-F	ACGGTCGCAAGACTGAAACT	
RT-16S rRNA-R	AAGGCACCAATCCATCTCTG	

a Underlined sequences represent restriction endonuclease sites.

### Site-directed mutagenesis and essentiality testing.

Site-directed mutagenesis and essentiality testing were performed as described previously (33). To obtain the *rpfB1* and *rpfB2* site-directed mutant strains, a mutation plasmid was constructed; for example, to obtain the Y213A mutation in RpfB1, the approximately 500-bp DNA fragments flanking the *L. enzymogenes rpfB1* gene were amplified with *Pfu* DNA polymerase using *L. enzymogenes* genomic DNA as the template and either *rpfB1* NdeI and *rpfB1* Y213A P1 (for the up *rpfB1* Y213A mutant) or *rpfB1* Y213A P1 and *rpfB1* HindIII (for the down *rpfB1* Y213A mutant) as the primers (Table 3). The fragments were connected by overlap PCR using the primers *rpfB1* NdeI and *rpfB1* HindIII. The fused fragment was digested with NdeI and HindIII and inserted into pBAD24M to obtain the plasmid pBAD24M-*rpfB1* Y213A. The other five site-directed mutant plasmids (T214A, G216A, T217A, G219A, and E361A) and *rpfB2* site-directed mutant plasmids were constructed using a similar method. These RpfB mutant plasmids were then introduced into E. coli strain JW1794 for the complementation study.

### Protein expression and purification.

Protein expression and purification were performed as described previously (35). To clone the *L. enzymogenes rpfB1* and *rpfB2* genes, genomic DNA extracted from *L. enzymogenes* was used for PCR amplification using *Pfu* DNA polymerase, and the primers are listed in Table 3. The PCR products were inserted into pET-28b (+) to produce the plasmids pET-*rpfB1* and pET-*rpfB1*. The *L. enzymogenes rpfB1* and *rpfB2* genes were verified by nucleotide sequencing by Genscript (Nanjing, Jiangsu, China). *rpfB1* and *rpfB2* with a vector-encoded His_6_-tagged N terminus were expressed in E. coli BL21(DE3) and purified with Ni-nitrilotriacetic acid (NTA) agarose (Qiagen, Chatsworth, CA, USA) using a nickel-ion affinity column (Qiagen). The protein purity was monitored by SDS-PAGE and matrix-assisted laser desorption ionization–time of flight (MALDI-TOF) mass spectrometry.

### Assay of acyl-CoA ligase activity.

The assay was performed as described previously (36). Briefly, the reaction buffer mixture contained 100 mM Tris-HCl (pH 7.2), 5 mM MgCl_2_, 5 mM ATP, 0.125 mM reduced CoA, and 0.5 mM fatty acid substrate. The total reaction volume was 500 μl and included 5 μg of purified protein. For the reaction, a mixture containing all of the components listed above (excluding CoA) was assembled, and 475 μl of the mixture was preincubated at 28°C for 3 min. The reaction was initiated through the addition of 25 μl of 5 mM reduced CoA (diluted to a final concentration of 0.5 mM), which was preincubated at 28°C for 3 min, rapidly mixed, and incubated at 28°C throughout the reaction. Immediately after mixing, a reading was obtained at the zero time point by removing 100 μl from the 500-μl reaction mixture, adding it to 100 μl of 0.4 mM 5,5′-dithiobis(2-nitrobenzoic acid) (DTNB; dissolved in 0.1 M potassium phosphate at pH 8.0), and measuring the absorbance at 412 nm. Subsequently, 100-μl aliquots of the reaction mixture were collected at 5-min intervals and mixed with DTNB to obtain additional measurements. The extinction coefficient of CoA was assumed to be 1.36 × 10^4^ M^−1 ^cm^−1^. All reactions involving RpfBs were repeated to obtain triplicate data for each fatty acid at each concentration. The maximum velocity (*V*_max_) of the enzymes and affinity for the different substrates (Michaelis constant, *K_m_*) were then determined using Hanes-Woolf plots.

### Deletion of *L. enzymogenes rpfB* genes and complementation.

To disrupt the *L. enzymogenes rpfB1* and *rpfB2* genes, the pEX18GM based suicide plasmids pEX18-Δ*rpfB1* and pEX18-Δ*rpfB2* were constructed for in-frame deletion. The approximately 500-bp DNA fragments flanking the *rpfB1* and *rpfB2* genes were amplified with *Pfu* DNA polymerase using *L. enzymogenes* genomic DNA as the template and either *rpfB1* HindIII and *rpfB1* up1 (for up *rpfB1*) and *rpfB1* down1 and *rpfB1* XbaI (for down *rpfB1*) or *rpfB2* HindIII and *rpfB2* up1 (for up *rpfB2*) and *rpfB2* down1 and *rpfB2* XbaI (for down *rpfB2*) as the primers (Table 3). The fragments were purified and joined by overlap PCR. The fused fragments were digested with HindIII and XbaI and inserted into pEX18GM (37) to obtain the plasmids pEX18-Δ*rpfB1* and pEX18-Δ*rpfB2*. The resulting constructs were transferred into *L. enzymogenes* by electroporation, and gentamicin was used to select for integration of the nonreplicating plasmid into the recipient chromosome. A single-crossover integrant colony was spread on LB medium without gentamicin and incubated at 28°C for 3 days, and after appropriate dilution, the culture was spread onto LB plates containing 15% sucrose. Colonies sensitive to gentamicin were screened by PCR using the primers listed in Table 3, and the *L. enzymogenes rpfB1* and *rpfB2* deletion strains (Δ*rpfB1* and Δ*rpfB2*) were obtained. For complementation of the *rpfB1* and *rpfB2* mutants, the coding regions of *rpfB1* and *rpfB2* were PCR amplified and cloned into the versatile pBBR1MCS5 plasmid (38). The resulting plasmids were transferred into the *L. enzymogenes* strain by electroporation and *rpfB1* and *rpfB2* complementation strains were obtained.

### Quantitative real-time PCR.

Quantitative real-time PCR was carried out according to previous studies (39). The bacterial cells were collected when the cellular optical density (OD_600_) reached 1.0 in 10% TSB. Total RNA was extracted using a TRIzol-based method (Life Technologies, CA, USA). RNA quality control was performed via the following steps: (i) the degree of RNA degradation and potential contamination were monitored on 1% agarose gels, (ii) the RNA purity (OD_260_/OD_280_, OD_260_/OD_230_) was checked using a NanoPhotometer spectrophotometer (Implen, CA, USA), and (iii) the RNA integrity was measured using the Bioanalyzer 2100 (Agilent, Santa Clara, CA, USA). The primers used in this assay are listed in Table 3. cDNA was then synthesized from each RNA sample (400 ng) using the TransScript all-in-one first-strand cDNA synthesis supermix for qPCR (one-step genomic DNA [gDNA] removal) kit (TransGen Biotech, Beijing, China) according to the manufacturer’s instructions. qRT-PCR was performed using the TransStart top green qPCR supermix (TransGen Biotech) on a QuantStudio TM 6 flex real-time PCR system (Applied Biosystems, Foster City, CA, USA) with the following thermal cycling parameters: denaturation at 94°C for 30 s, followed by 40 cycles of 94°C for 5 s, and 60°C for 34 s. Gene expression analyses were performed using the 2^–ΔΔ^*^CT^* method with the 16S rRNA gene as the endogenous control, and the expression level in the wild type was set to a value of 1. The experiments were performed three times, and three replicates were examined in each run.

### Bacterial one-hybrid assays.

Bacterial one-hybrid assays were performed as previously described (40, 41). In brief, the bacterial one-hybrid reporter system contains three components, namely, the plasmids pBXcmT and pTRG, which are used to clone the target DNA and to express the target protein, respectively; and the E. coli XL1-Blue MRF′ kan strain, which is the host strain for the propagation of pBXcmT and pTRG recombinants (42). In this study, the *L. enzymogenes rpfB1* (451 bp) and *rpfB2* (486 bp) promoter regions were cloned into pBXcmT to generate the recombinant vectors pBXcmT-p*rpfB1* and pBXcmT-p*rpfB2*, respectively. Similarly, the coding region of Clp (690 bp) was cloned into pTRG to create the final construct pTRG-Clp. The two recombinant vectors were transformed into the XL1-Blue MRF′ kan strain. If direct physical binding occurred between Clp and the *L. enzymogenes rpfB1* or *rpfB2* promoter, the positive-transformant E. coli strain containing both pBXcmT-p*rpfB1* and pTRG-Clp or both pBXcmT-p*rpfB2* and pTRG-Clp would grow well on selective medium, that is, minimal medium containing 5 mM 3-amino-1,2,4-triazole, 8 μg/ml streptomycin, 12.5 μg/ml tetracycline, 34 μg/ml chloramphenicol, and 30 μg/ml kanamycin. Furthermore, cotransformants containing pBX-R2031/pTRG-R3133 served as a positive control (42), and cotransformants containing empty pTRG and either pBXcmT-p*rpfB1* or pBXcmT-p*rpfB2* were used as a negative control. All cotransformants were spotted onto selective medium, grown at 28°C for 3 to 4 days, and then photographed.

### Electrophoretic mobility gel shift assays.

EMSA was performed as previously described (43, 44). For *L. enzymogenes* Clp gel shift assays, we used DNA fragments that included the *L. enzymogenes rpfB1* (451 bp) or *rpfB2* (486 bp) promoter region as a probe. The probe DNA (50 ng) was mixed with protein in a 20-μl reaction mixture containing 10 mM Tris (pH 7.5), 50 mM KCl, 1 mM dithiothreitol, and 0.4% glycerol. After incubation for 30 min at 28°C, samples were electrophoresed on a 5% nondenaturing acrylamide gel in 0.5× Tris-borate-EDTA (TBE) buffer at 4°C. The gel was soaked in 10,000-fold-diluted SYBR green I nucleic acid dye (Sangon Biotech, Shanghai, China), and the DNA was visualized at 300 nm.

### HSAF extraction and quantification.

HSAF was extracted from 50-ml *L. enzymogenes* cultures grown in 10% TSB for 48 h at 28°C with shaking (at 180 rpm). HSAF was detected via HPLC and quantified per unit of OD_600_ as described previously (10, 20, 31). Three biological replicates were used, and each was examined with three technical replicates.

### Detection of DSF signal components in the *L. enzymogenes* culture supernatant.

The protocols used for the extraction and purification of DSF family components were described previously (12). *L. enzymogenes* strains were cultured in liquid medium for 24 h, and 50 ml of the bacterial supernatant was collected by centrifugation at 4000 × *g* and 4°C for 15 min. The pH of the supernatants was adjusted to 4.0 by adding hydrochloric acid prior to two extractions with an equal volume of ethyl acetate. Ethyl acetate fractions were collected, and the solvent was removed by rotary evaporation to dryness at 42°C. The residue was dissolved in 100 μl of methanol. Crude extract was subjected to 0.22-μm Mini-Star filtration, and the filtrate was concentrated to 100 μl for liquid chromatography-tandem mass spectrometry (LC-MS/MS) analysis. The extract (2 μl) was injected into a C_18_ reversed-phase column (2.1 × 100 mm; Agilent Poroshell SB-C_18_) and eluted with water in methanol (20:80 [vol/vol]) at a flow rate of 0.3 ml/minute in a QTRAP 6500 LC-MS/MS system (AB Sciex, USA).

### Statistical analyses.

The experimental data sets were subjected to analyses of variance using GraphPad Prism 7.0. The significance of the treatment effects was determined by the *F* value (*P* = 0.05). If a significant *F* value was obtained, a separation of means was accomplished by Fisher’s protected least significant difference at a *P* value of ≤0.05.

### Data availability.

The data that support the findings of this study are openly available in GenBank under accession numbers RCTY01000033 (Lysobacter enzymogenes strain OH11 scffold34, whole-genome shotgun sequence; Le4724/Le RpfB1, and locus tag D9T17_13860) and RCTY01000001 (Lysobacter enzymogenes strain OH11 scffold1, whole-genome shotgun sequence, Le2453/Le RpfB2, locus tag D9T17_00210).

## Supplementary Material

Supplemental file 1
