# Supplementary material for: Two Functional Fatty Acyl Coenzyme A Ligases Affect Free Fatty Acid Metabolism To Block Biosynthesis of an Antifungal Antibiotic in Lysobacter enzymogenes
Source: Appl Environ Microbiol. 2020 May 5;86(10):e00309-20. doi: 10.1128/AEM.00309-20 (PMC7205486; doi:10.1128/AEM.00309-20)
Supplement: Supplemental file 1 [file AEM.00309-20-s0001.pdf]

Supplemental Material

Two functional acyl-CoA ligases affect free fatty acid metabolism to block biosynthesis of an antifungal antibiotic in *Lysobacter enzymogenes*

Kaihuai Li[1], Rongxian Hou[1], Huiyong Xu[2], Guichun Wu[2], Guoliang Qian[1], Haihong Wang[3], Fengquan Liu[1, 2, *]

Contents

Fig. S1

Fig. S2

Fig. S3

Fig. S4

Fig. S5

Fig. S1

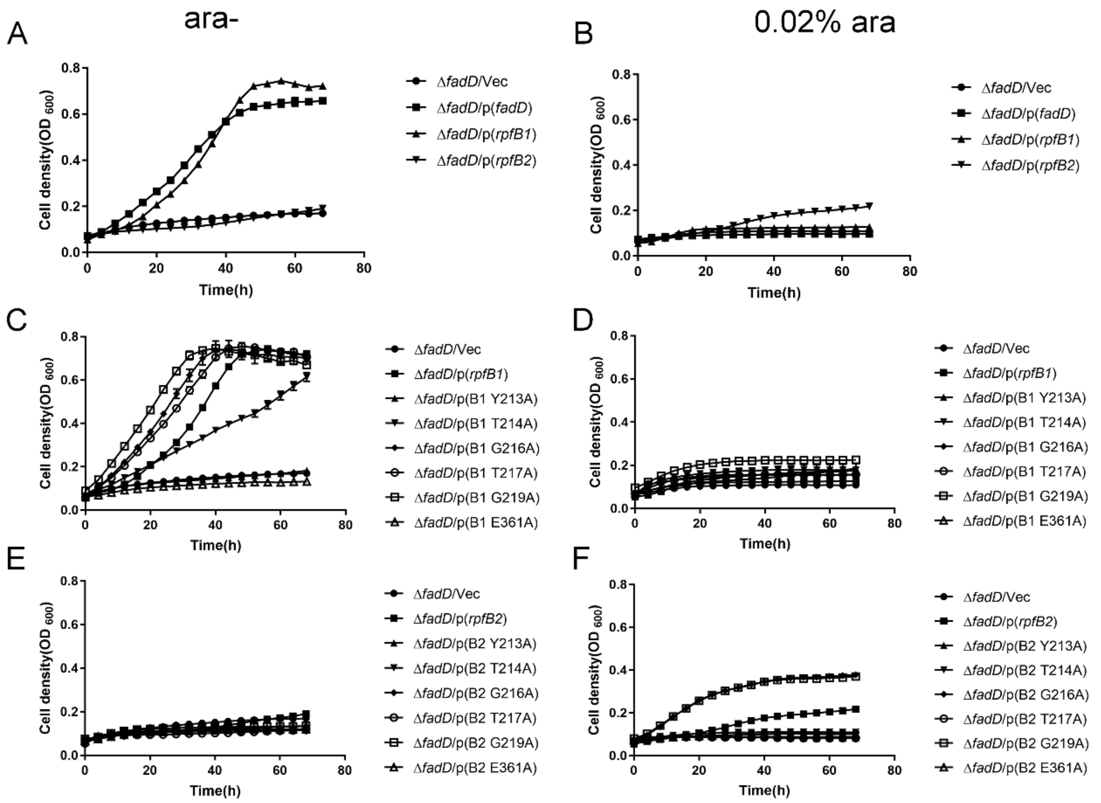

Fig. S1. Growth curve of JW1794 transformants grown in M9 broth with oleic acid as the sole carbon source. Growth was tested in either the presence or absence of arabinose. The strains tested were the following: **(A-B)** JW1794 carrying the plasmid pBAD24M-EcD, pBAD24M-*rpfB1*, or pBAD24M-*rpfB2*, encoding *E. coli fadD* (EcfadD), Le *rpfB1* or Le *rpfB2*, respectively, or the vector plasmid pBAD24M. **(C-D)** Strain JW1794 carrying the plasmid pBAD24M-*rpfB1*, pBAD24M-*rpfB1* Y213A, pBAD24M-*rpfB1* T214A, pBAD24M-*rpfB1* G216A, pBAD24M-*rpfB1* T217A, pBAD24M-*rpfB1* G219A or pBAD24M-*rpfB1* E361A, encoding Le *rpfB1* or the, Y213A, T214A, G216A, T217A, G219A or E361A *rpfB1* mutant, respectively, or the vector plasmid pBAD24M. **(E-F)** Strain JW1794 carrying the plasmid pBAD24M-*rpfB2*, pBAD24M-*rpfB2* Y213A, pBAD24M-*rpfB2* T214A, pBAD24M-*rpfB2* G216A, pBAD24M-*rpfB2* T217A, pBAD24M-*rpfB2* G219A or pBAD24M-*rpfB2* E361A, encoding Le *rpfB2* or the, Y213A, T214A, G216A, T217A, G219A or E361A *rpfB2* mutant, respectively, or the vector plasmid, pBAD24M.

Fig. S2

A

Le *rpfB1* Promoter, Predicted Le Clp Binding Site

CGCGGTCGATGCCGGAGCCGAGCCCGGCCGCTGCCGATCCTGACA
G…142 bp…ATGAGTTTGAACCGTCCGTGGC

5´- ATGC-N22-CGAT - 3´

B

Le *rpfB2* Promoter, Predicted Le Clp Binding Site

ATACCGCAATCGCGGTCAAAGAATGGATGGCGTCCGATTCGGCTTC
…41 bp…GTGGAGCGCGCCGGCTTGCT

5´- ATCG-N22-GCAT - 3´

Fig. S2. Clp binding to sites upstream of Le *rpfB1* and Le *rpfB2*. (A) DNA sequence of the region upstream of Le *rpfB1* showing putative Clp-binding sites (underlined). **(B)** DNA sequence of the region upstream of Le r*pfB2* showing putative Clp-binding sites (underlined).

Fig. S3

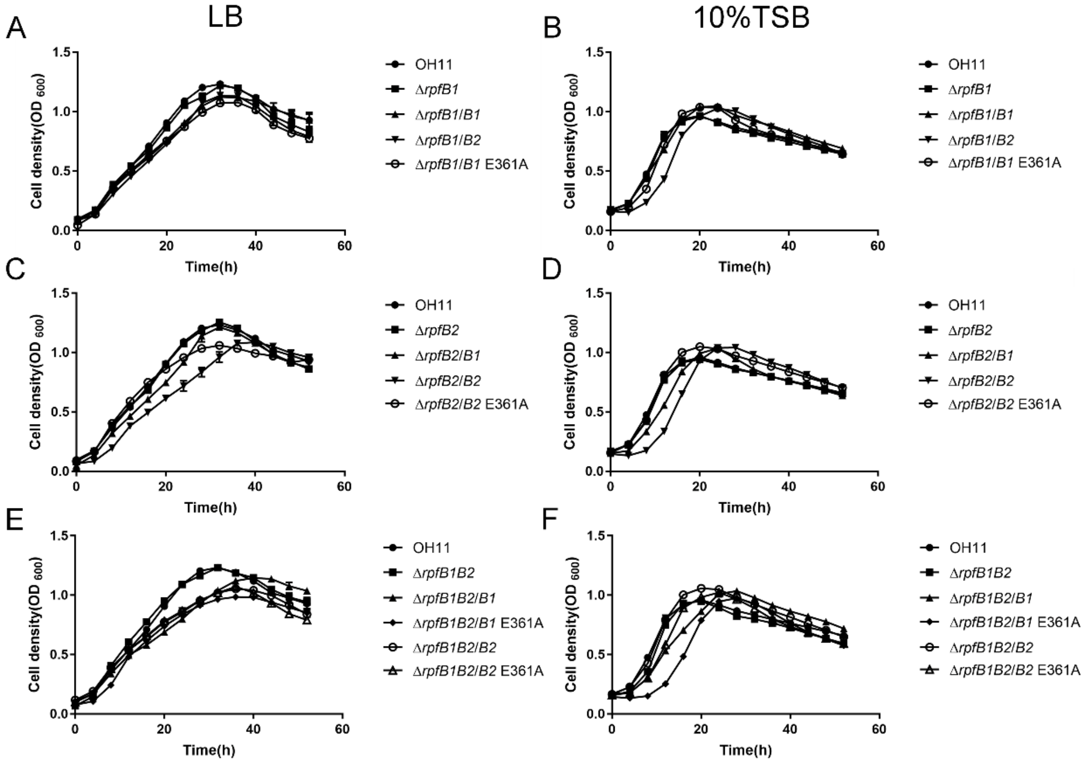

Fig. S3. Le *rpfB* mutants exhibited no significant difference in bacterial density. **(A)** Growth curves of the *rpfB1* mutant strain and strains complemented with the *rpfB1* or the *rpfB1* site-directed mutant gene in rich LB medium. **(B)** Growth curves of the *rpfB1* mutant strain and strains complemented with the r*pfB1* or the *rpfB1* site-directed mutant genes in 10% TSB medium. **(C)** Growth curves of the *rpfB2* mutant and of the strains complemented with *rpfB2* or the *rpfB2* site-directed mutant gene in rich LB medium. **(D)** Growth curves of the *rpfB2* mutant strain and strains complemented with the *rpfB2* gene or *rpfB2* site-directed mutant gene in 10% TSB medium. **(E)** Growth curves of the *rpfB1B2* double-mutant strain and strains complemented with the *rpfB1* gene, *rpfB2* gene, *rpfB1* site-directed mutant gene or *rpfB2* site-directed mutant gene in rich LB medium. **(F)** Growth curves of the *rpfB1B2* double-mutant strain and strains complemented with the *rpfB1* gene, *rpfB2* gene, *rpfB1* site-directed mutant gene or *rpfB2* site-directed mutant gene in 10% TSB medium.

Fig. S4

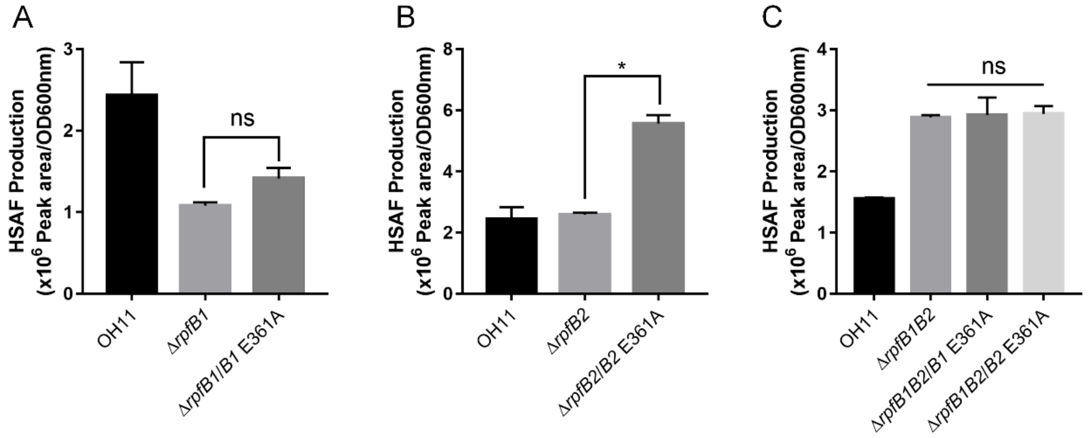

Fig. S4. Residue Glu-361 in RpfB1 and RpfB2 was essential for blocking HSAF biosynthesis. (A) Quantification of HSAF in the *rpfB1* mutant strains complemented with the *rpfB1* site-directed mutant gene. **(B)** Quantification of HSAF in the *rpfB2* mutant strains complemented with the *rpfB2* site-directed mutant gene. **(C)** Quantification of HSAF in the *rpfB1B2* double mutant strains complemented with the *rpfB1* site-directed mutant gene or *rpfB2* site-directed mutant gene. Error bars, means ± standard deviations (n = 3). ∗ $P < 0.05$, assessed by one-way ANOVA. All experiments were repeated three times with similar results.

Fig. S5

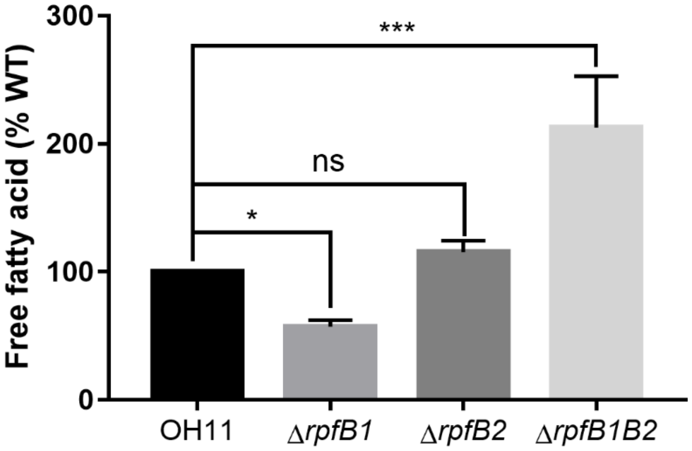

Fig. S5. Quantification of the cellular fatty acid content in the Δ*rpfB1,* Δ*rpfB2* and Δ*rpfB1B2* mutant strains. Error bars, means ± standard deviations (n = 3). ∗ P < 0.05, ∗∗∗ P < 0.001, assessed by one-way ANOVA. All experiments were repeated three times with similar results.
